# Flexor tendon injuries following plate fixation of distal radius fractures: a systematic review of the literature

**DOI:** 10.1007/s10195-013-0245-z

**Published:** 2013-05-14

**Authors:** Saeed Asadollahi, Prue P. A. Keith

**Affiliations:** Department of Orthopaedics, North East Health Wangaratta, 23 Green St, Wangaratta, VIC 3677 Australia

**Keywords:** Flexor tendon rupture, Volar plate fixation, Distal radius fracture, Flexor pollicis longus, Flexor digitorum profundus, Watershed line

## Abstract

**Background:**

Flexor tendon rupture is a rare but major complication associated with volar plate fixation of distal radius fractures.

**Materials and methods:**

We performed a systematic review to evaluate the demographics, clinical profile, treatment and outcome of flexor tendon rupture following volar plate fixation of distal radius fracture. Electronic searches of the MEDLINE, EMBASE, and Cochrane databases for systematic reviews and conference proceedings were performed. Studies were included if they reported flexor tendon rupture (partial or complete) as a complication of distal radius fracture plating (all levels of evidence).

**Result:**

Our search yielded 21 studies. There were 12 case reports and 9 clinical studies. A total of 47 cases were reported. There were 11 males and 23 females (*n* = 16 studies). The mean age was 61 years old (range 30–85). The median interval between the surgery and flexor tendon rupture was 9 months (interquartile range, 6–26 months). Twenty-nine plates were locking and 15 were nonlocking (*n* = 20 studies). FPL was the most commonly ruptured tendon (*n* = 27 cases, 57 %), with FDP to index finger being the second most common (*n* = 7 cases, 15 %). Palmaris longus tendon graft and primary end-to-end repair were the most common surgical methods used in cases of FPL tendon rupture.

**Conclusion:**

Flexor tendon rupture is a recognised complication of volar plating of distal radius fracture. Positioning of the plate proximal to the “watershed” line and early removal of the plate in cases with plate prominence or warning symptoms can reduce the risk of this complication.

## Introduction

Distal radius fractures are common orthopaedic injuries comprising 8–17 % of all extremity fractures and as many as 72 % of all forearm fractures [1–4], with an incidence of 26 per 10,000 person-years [5, 6]. Internal fixation with volar locking plates is becoming an increasingly popular technique for management of displaced and/or unstable distal radius fractures [5, 7–9]. This technique has the theoretical advantage of reducing tendon irritation, a complication associated with dorsal plate fixation [5].

However, a risk of flexor tendon irritation is associated with volar internal fixation [7, 10–12]. Soong et al. [13] reported flexor tendon irritation as the most common late complication, affecting 4.3 % of patients in their series. Several authors have described flexor tendon complications (i.e. partial or complete rupture, flexor tenosynovitis) following volar plating of distal radius fractures [10, 14–21]. The likely contributing factor is tendon wear over the edge of a prominent plate [8, 11, 17, 22–24].

The objective of this study was to search the literature and determine the demographics, clinical profile, treatment method (type of plate) and outcome of flexor tendon rupture following volar plate fixation of distal radius fracture. To our knowledge this is the most comprehensive review of flexor tendon injury associated with volar plate fixation in the English language literature.

## Materials and method

In November 2011, an electronic search of MEDLINE (1950–present) (via PubMed) and EMBASE was performed. The Cochrane Database of Systematic Reviews (CDSR) and the conference proceedings of 2011 for the Australian Orthopaedic Association (AOA), American Academy of Orthopaedic Surgeons (AAOS), British Orthopaedic Association (BOA), and Canadian Orthopaedic Association (COA) were also searched electronically. The search terms used were as follows: “flexor tendon rupture”, “radius fracture”, “plate”, “internal fixation” and “complication”. Reference sections of all accessed papers were searched for any undetected studies. English language restriction was applied. The studies were shortlisted for inclusion if they pertained to flexor tendon complications after volar plate fixation of distal radius fractures. The abstracts of the shortlisted studies were then reviewed and selected abstracts were considered for the full text review. Studies were included if they reported flexor tendon rupture (partial or complete) as a complication of distal radius fracture plating (all levels of evidence). When a study was selected, the following data were extracted: patient age, sex, interval between the operation and flexor tendon rupture, type of plate used, patients’ symptoms, cause of rupture from the authors’ perspective, treatment and outcome.

Statistical analysis was performed with SPSS (version 16; SPSS, Chicago, IL, USA). Studies that were included for analysis contained individual patient data or summary data with means and/or ranges of demographics. Simple descriptive statistics and frequency analyses were performed on multiple variables. The data are presented as median and interquartile ranges when they are not normally distributed.

## Results

**Fig. 1:**
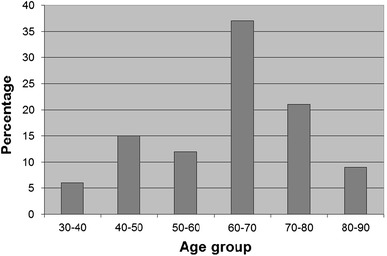
Bar chart demonstrating the age distribution of the patients with flexor tendon rupture following volar plate fixation of distal radius fracture

**Fig. 2:**
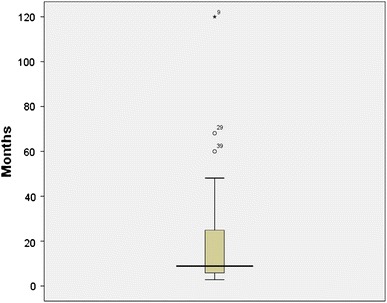
Box plot of the interval between the surgery and flexor tendon rupture

We found 21 studies reporting flexor tendon rupture as a complication of volar plating of distal radius fracture (Table 1). There were 12 case reports and 9 clinical studies (level III evidence) in which the flexor tendon rupture was reported as part of the study. Most of the reports were published after 2006 (77 %). A total of 47 cases were reported. There were 11 males and 23 females (*n* = 16 studies). The mean age of these patients was 61 years old (range 30–85). The age distribution of the patients is shown in Fig. 1. The median interval between the surgery and flexor tendon rupture was 9 months (*n* = 39, interquartile range, 6–26 months) (Fig. 2). Flexor tendon rupture was reported as early as 3 months [21] and as late as 10 years after volar plating [25]. Twenty-nine plates were locking and 15 were nonlocking (*n* = 20 studies). Flexor pollicis longus (FPL) was the most commonly ruptured tendon (*n* = 27 cases, 57 %) with the flexor digitorum profundus (FDP) to the index finger being the second most common (*n* = 7 cases, 15 %). Two or more flexor tendon ruptures occurred in 5 cases (10 %).

**Table 1:** Literature review showing cases of flexor tendon rupture following volar plate fixation of distal radius fracture

Reference	Years	Level of evidence	No. of cases	Age (years)	Ruptured tendon	Sex (M/F)	Delay to rupture (months)	Type of plate	Plate manufacturer
Present work^a^	2013	IV	1	75	FPL FDP I	1/0	6	Volar locking plate	Synthes
Yangyang et al. [36]	2011	III	1	–^b^	FPL	–	–	Volar locking plate	–
Minegishi et al. [20]	2011	III	1	–	FPL	–	–	Volar locking plate	Acumed
Soong et al. [13]	2011	III	1	–	FPL	–	8	–	–
Soong et al. [10]	2011	III	3	79	FPL FDP I^c^ FDP I and FDS I^d^	1/2	20	Volar locking plate	Acumed
Brown and Lifchez [23]	2011	IV	1	75	FPL (partial rupture)	0/1	30	Volar locking plate	Acumed
Figl et al. [38]	2010	III	1	–	FPL	–	4	Volar locking plate	Midartis
Lifchez [26]	2010	IV	1	65	FPL	0/1	10	Volar locking plate	–
Monda et al. [25]	2010	IV	1	30	FPL	1/0	120	Nonlocking buttress plate	–
Valbuena et al. [12]	2010	IV	5	56	FPL (3) FDP I and FDP M (1) FDP I (1)	3/2	44	Nonlocking buttress plate	–
Casaletto et al. [11]	2009	IV	7	62	FPL	3/4	13	Volar locking plate	Acumed (*n* = 4) Synthes (*n* = 3)
Adham et al. [29]	2009	IV	3	60	FDP I and FDS I FDP M and FDS M^e^ (partial) FPL and partial rupture of FCR Partial rupture of FPL	0/3	5	Volar locking plate	Hand Innovations KMI Viper plate Acumed
Cross and Schmidt [22]	2008	IV	1	51	FPL	0/1	6	Volar locking plate	Acumed
Klug et al. [27]	2007	IV	1	59	FPL	0/1	13	Volar locking plate	Synthes
Arora et al. [8]	2007	III	2	–	FPL	–	–	Volar locking plate	Synthes
Rampoldi and Marsico [28]	2007	III	1	48	FPL	1/0	8	Volar locking plate	Synthes
Duncan et al. [16]	2007	IV	1	Late 70s	FPL	0/1	9	Nonlocking buttress plate	Synthes
Koo and Ho [15]	2006	IV	1	63	FPL	0/1	68	Nonlocking buttress plate	Synthes
Drobetz and Kutscha-Lissberg [21]	2003	III	6	–	FPL	–	10	Volar locking plate	Mathys
Nunley and Rowan [14]	1999	IV	1	72	FPL	0/1	10	Nonlocking plate	Synthes
Bell and Wollstein [17]	1998	III	4	67	3 FPL 1 FPL partial rupture	0/4	7	Nonlocking AO buttress plate	–
Fuller [18]	1973	IV	2	Mean 47.5	FPL FDP I	1/1	33 (6–60)	Nonlocking volar plate (Ellis)	–

^a^Case presented in this paper

^b^Extractable data not available

^c^Flexor digitorum profundus to the index finger

^d^Flexor digitorum superficialis to the index finger

^e^The flexor digitorum profundus and flexor digitorum superficialis tendons to the long finger

**Fig. 3:**
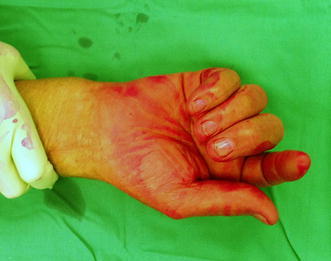
Seventy-five-year-old male with FPL and FDP to index finger ruptures following volar plating of distal radius fracture

Patients presented symptoms including an inability to flex the interphalangeal joint of the affected phalanx (Fig. 3) associated with pain and a rubbing sensation with movement [12, 22, 26–28], sometimes preceded by volar, radial-sided wrist pain [16, 22, 26], volar wrist swelling, and a pop or clicking sensation [17, 29]. The causes of flexor tendon rupture were excessive distal placement of the plate [10, 17, 18, 20, 22], a prominent distal edge (initial lift-off or as a result of fracture collapse) [12, 15–17, 28, 29], palmarly protruding screws [8, 21, 29], and dorsal malunion of the distal radius [21, 25].

Table 2 shows the details of the treatment for flexor tendon rupture after distal radius volar plating (*n* = 32). Palmaris longus tendon graft and primary end-to-end repair were the most common modes of management in cases of FPL tendon rupture. A few reports presented the detailed outcome of surgical treatment for flexor tendon rupture [12, 14–16, 22]. Out of 8 cases who had operative treatment of the FPL rupture and documented the postoperative range of movement of the thumb interphalangeal joint, only 2 cases achieved a full range of movement (one after end-to-end repair, and one following palmaris longus tendon transfer) [12, 14–16, 22].

**Table 2:** Systematic review of management of flexor tendon rupture after distal radius volar plating

Tendon ruptured	Treatment						
	End-to-end repair	FPL tenodesis	Palmaris longus tendon graft	Tendon graft	Tendon transfer	Thumb IP joint fusion	Total
FPL	8	1	9	1	4	2	25
FDP to IF	1	0	0	2	1	0	4
FDP to MF	1	0	0	0	0	0	1
FDS to IF	0	0	0	1	0	0	1
FDS to MF	0	0	0	1	0	0	1
Total	10	1	9	5	5	2	32

### Case example

A 75-year-old right-handed farmer sustained bilateral comminuted intra-articular distal radial fractures following a fall from a utility vehicle. He underwent open reduction and internal fixation (ORIF) of both distal radius fractures using volar fixed-angle locking plates (Synthes, Paoli, PA, USA). Six months postoperatively, he was referred to the senior author with a 4-week history of inability to flex the interphalangeal joint of the right thumb and a 1-week history of inability to flex the distal interphalangeal joint of the index finger. There were no relevant risk factors in his medical or pharmaceutical history that predisposed him to tendon rupture, and he was a nonsmoker. The patient consented to publication of his case.

Examination revealed an inability to flex the interphalangeal joint of the right thumb and distal interphalangeal joint of the right index finger. No other deficits were noted. The surgical scar was well healed.

**Fig. 4:**
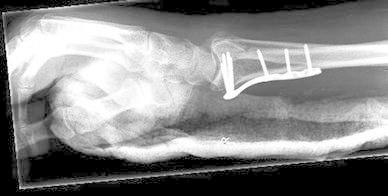
Lateral X-ray showing a slightly prominent distal edge of the plate

**Fig. 5:**
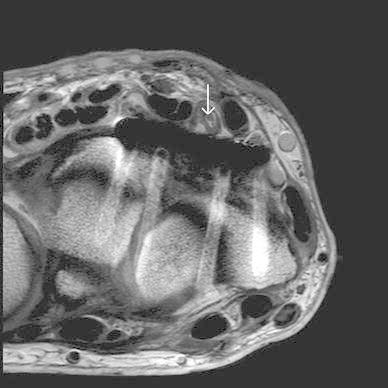
MRI showing FPL rupture (*white arrow*) and close proximity of the flexor tendon to the volar plate (MRI performed before FDP to index finger rupture)

The X-ray showed a united fracture in a satisfactory position. Review of the immediate postoperative X-ray showed the plate was prominent volarward, with some lift-off (Fig. 4). An MRI scan confirmed the FPL rupture (Fig. 5).

**Fig. 6:**
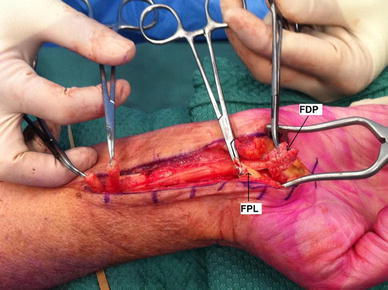
Intraoperative picture showing the frayed ends of the ruptured FDP and FPL

The patient was taken to surgery for immediate exploration to address the tendon ruptures. A midpalmar approach was utilised and extended distally to the carpal tunnel. Complete ruptures of the FPL and FDP to index finger were noted. The proximal stump of the FDP was retracted approximately 4–5 cm. Both tendons were substantially frayed and were thickened and synovitic throughout (Fig. 6). There was gross flexor synovitis at the level of the wrist. The plate was removed and flexor tenosynovectomy performed. Direct end-to-end repair of the FDP tendon was performed. The FPL tendon was repaired using the ipsilateral palmaris longus tendon graft. The carpal tunnel was decompressed.

**Fig. 7:**
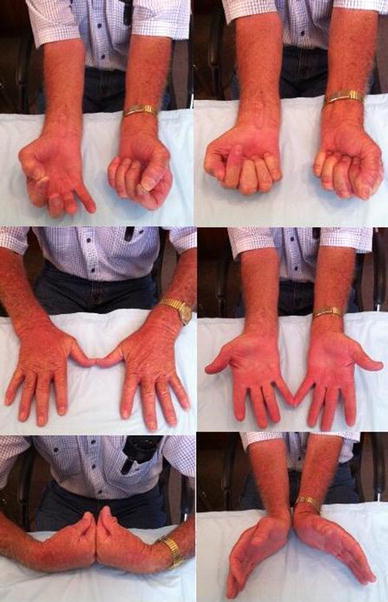
Functional outcome at the 14-month follow-up

At the 14-month follow-up visit, he had full flexion of the interphalangeal joint of his right thumb. The patient had full passive flexion but no active flexion of the distal interphalangeal joint of the right index finger (Fig. 7). The active right wrist range of movement was 20° flexion, 20° extension, 15° of ulnar deviation, and 20° of radial deviation. The range of movement of the left wrist was 35° flexion, 35° extension, 30° of ulnar deviation, and 30° of radial deviation.

The grip strength was 23 kg on the right and 32 kg on the left (Jamar hand dynamometer, North Coast Medical, Morgan Hill, CA, USA). The right index finger strength was 2 kg and the left index finger strength was 10 kg. The Disabilities of the Arm, Shoulder and Hand (DASH) [30] score was 8.8. Wrightington Wrist Function Score was 13 for the right wrist and 8 for the left wrist (range 8–32, with 8 being the best possible score) [31].

## Discussion

Volar fixed-angle plating of distal radius fractures was developed as an alternative to dorsal plate fixation [8, 22, 29]. The perceived advantages of volar plating include a familiar and straightforward surgical approach [15, 32], a theoretical advantage in reducing the complication of tendon attrition due to an absence of flexor tendon–bone intimacy [5], the use of a thicker and stronger implant to better resist load [33], and the lack of a need for routine plate removal [5].

Flexor tendon rupture is a major complication after volar plating of distal radius fractures, and our review of the literature shows 47 reported cases. This likely represents only a portion of the real number. This review demonstrates that flexor tendon rupture can occur with various plate designs, including the new-generation volar locking plates [23, 26]. Surgeons should be aware of this complication. Advising and monitoring the patient are recommended. Fifty percent of the reported cases in the literature occurred within 6–26 months after the operation. This signifies that, at the last follow-up visit, it is essential for patients to have the warning symptoms of flexor tendon irritation [34] explained to them and to be advised to see the surgeon as soon as they appear.

Our systematic review shows that more than one-third of the population (37 %) who are reported to have a flexor tendon rupture are between 60 and 70 years old, and women are affected almost twice as often (female to male ratio: 23/11). This can be explained by a higher incidence of distal radius fracture amongst women secondary to osteoporosis with increasing age [6]. The decrease in the tensile properties of the tendon with age may also make it more susceptible to attritional wear against the prominent plate edge [35]. The incidence of distal radius fracture also increases with age. However, as shown in this review, the incidence of flexor tendon rupture tends to decrease after the age of 70, which could be because distal radius fractures are probably managed by alternative treatment options (i.e. closed reduction, pinning) with increasing age, and therefore fewer complications are reported in the literature in this group [6].

**Fig. 8:**
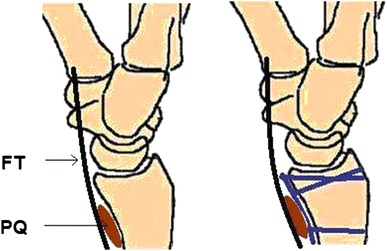
Schematic presentation of the watershed line and flexor tendon impingement over a prominent distal plate edge. *PQ* pronator quadratus, *FT* flexor tendon

The pathogenesis of flexor tendon rupture after volar plating of distal radius fracture is multi-factorial and includes: excessive distal placement of the plate [11, 17, 18], prominent distal edge of the plate [12, 16, 17, 29, 36], prominent screw heads [8, 21], inbuilt malreduction, incorrect plate usage [10, 16], fracture collapse [17], tendinopathy for any reason (i.e. steroid use) [17], and iatrogenic injury [16]. Plate positioning has been suggested to be one of the main contributing factors to flexor tendon irritation after volar plating of distal radius fractures [8, 22]. The “watershed” line is a transverse ridge that lies distal to the pronator quadratus muscle and is located within 2 mm of the joint line on the ulnar side of the radius, 10–15 mm from the articular surface on the radial side of the bone [37]. A volar plate that is placed distal to the watershed line can potentially impinge on the traversing flexor tendons, resulting in irritation or rupture (Fig. 8) [5, 37]. In a recent cadaveric study, Tanaka et al. showed that at 30° or 60° of wrist extension, contact pressure between the distal plate edge and the FPL tendon significantly increased when the distal plate edge was placed distal to the watershed line compared with when it was placed proximal to or at the watershed line. In the authors’ case example, the prominent distal edge of the plate was the likely cause of tendon rupture. Even though the plate was placed proximal to the watershed line, it was still prominent volarward, and likely reflected an inbuilt malreduction of the fracture as well as the inbuilt plate features. We believe that the use of a low-profile plate designed for more proximal placement could have decreased the possibility of this complication.

Various strategies have been suggested to minimise the chance of flexor tendon attrition following volar plate fixation of distal radius fractures [5, 24]. These include placement of the plate on or proximal to the watershed line [5, 37], repair of the pronator quadratus muscle, and early removal of the plate. However, correct positioning of a volar plate for fixation of a distal radial fracture is dictated in part by the fracture pattern, the plate design and the manufacturers’ recommendations [27], and the surgical experience of the operator. In practice, placement of a plate that conforms to the volar anatomy of the distal radius proximal to the “watershed” line may not always be possible [16]. For example, sometimes the plate has to be placed very distally to provide a subchondral buttress to the palmar aspect of the articular surface or to stabilize very distal fracture types [8]. Ideally, these situations should be avoided by choosing a suitable implant design and preoperative planning. If the plate has to be seated more distally, then close monitoring of these patients postoperatively and removal of the plate at the first sign of flexor tendon irritation should be considered [16, 21, 23]. Our experience of FPL and FDP to index finger ruptures occurring 3 weeks apart shows that it is probably essential to react quickly to these complaints and intervene before complete tendon rupture follows [23].

The pronator quadratus muscle covers the volar surface of the distal radius and provides protection for the flexor tendons from deeper structures such as prominent hardware [10, 17]. However, repair of the pronator quadratus muscle may not always be the solution to the issue of flexor tendon attrition, as the quality of muscle is not always achievable or optimal [32]. Pronator quadratus muscle was not repaired in the authors’ case, which may have contributed to flexor tendinitis and rupture.

The systematic review in this study has some limitations. The main body of the literature from which the information was extracted does not have a high quality of evidence (level III and IV studies). In many instances, not all of the details of the cases were reported (i.e. demographics, type of plate, management of complication, outcome), which limits the number of extractable variables and the quality of analysis.

Flexor tendon rupture is an uncommon yet serious complication of volar plate fixation of distal radius fracture that exists despite recent advances in plate design. Appropriate plate selection and careful surgical technique are necessary to prevent this complication. Patient consent should ideally include this potential complication given its implications, and patients should be advised at follow-up to report any increase in pain, crepitus or change in thumb or finger motion. Surgeons should be aware of this complication and react appropriately to prevent possible complete rupture of tendons.
